# Online users’ donation behavior to medical crowdfunding projects: Mediating analysis of social presence and perceived differences in trust

**DOI:** 10.3389/fpsyg.2022.1008494

**Published:** 2022-09-28

**Authors:** Tao Zhang, Qianyu Zhang, Rong Jiang, Tilei Gao, Ming Yang

**Affiliations:** ^1^School of Information, Yunnan University of Finance and Economics, Kunming, Yunnan, China; ^2^Intelligent Application Research Institute, Yunnan University of Finance and Economics, Kunming, Yunnan, China

**Keywords:** cognition, perception, perceived trust, perceived differences, social presence, medical crowdfunding, donation behavior

## Abstract

Perceived trust is a key factor affecting the behavior to donate online. In order to further explore the factors and influencing mechanisms that affect the success of medical crowdfunding projects, this paper, combined with the Stimulus-Organism-Response (S-O-R) theory, introduces the mediating variable of social presence and perceived differences in trust, and constructs a model of online users’ donation behavior to medical crowdfunding projects. We collected 437 valid samples through a questionnaire survey, and processed the data with SPSS and Amos software to test and analyze the theoretical model. The research results showed that project description and user participation have a significant positive effect on social presence; project transparency and patient identity have a significant negative effect on perceived differences in trust; social presence has a positive effect on donation behavior, while the perceived difference in trust has a negative effect; social presence and perceived differences in trust play a mediating role respectively; there is no significant effect of patient status on social presence. This study further expands the application of social presence and perceived differences in trust in medical crowdfunding, and provides a theoretical basis for the success of medical crowdfunding projects.

## Introduction

Compared with the traditional public welfare crowdfunding, the medical crowdfunding appeared under the internet background not only has the functions of traditional charity to help people in need but also has the advantages of mobilizing friends around to raise funds in a short time in terms of fundraising ability (Chin et al., 2021a). The emergence of these platforms has made crowdfunding increasingly common to raise medical expenses. Due to limited medical resources and differences between urban and rural public medical systems, medical crowdfunding projects can play a complementary role in supporting the current public medical insurance system (Ba et al., 2021). Therefore, studying the factors and influencing mechanisms that affect the success of medical crowdfunding projects has become a research issue that cannot be ignored in academic circles.

Currently, users mostly use social media for medical crowdfunding, connecting people with unmet medical needs with charity donors (Kubheka, 2020). Only by deeply analyzing the factors of online users’ donation behavior to medical crowdfunding projects, and further exploring the important factors and influencing mechanisms that affect the success of crowdfunding projects from the perspective of participants, can it be helpful to improve users’ enthusiasm for participating in medical crowdfunding project donations and guide more potential donations, which is of great significance to promoting the success of public welfare and medical crowdfunding projects. Previous studies have mostly focused on the direct influencing factors of the behavior to donate medical crowdfunding projects (Zhang et al., 2021a; Zhao et al., 2021), mainly including project description, project transparency, patient status, patient identity, user participation, etc., but there is a relative lack of research on the intermediary effect of the behavior.

Given the above background and problems, this paper constructs an intermediary effect model through the social presence and perceived differences in trust which can identify the motivation of donation from a cognitive perspective, and deeply studies the influence mechanism in donation behavior of medical crowdfunding projects. Through empirical analysis, this paper explores the influence mechanism of online users on the donation behavior of medical crowdfunding projects and verifies the mediating roles of the social presence and perceived differences in trust, and then provides the practical theoretical basis and practical guidance for stimulating online users’ donation behavior and enhancing the vitality of the crowdfunding industry.

This paper can be divided into the following parts: In section 1, the background, content, and significance of the research are presented; section 2 reviews relevant theories such as social presence, perceived differences in trust and S-O-R theory, and puts forward the research hypothesis and constructs the research model; section 3 entails the details about the “Research Methods,” especially designs the scale; in section 4, the hypothesis test and mediation effect test are carried out; and finally in section 5, the research is summarized, and the future research direction is pinpointed.

## Theory and hypotheses

### Social presence

Social presence was first proposed by researchers such as Professor Short at the University of Maryland (Short et al., 1976), who extended the social presence to related research in the field of electronic communication, and defined it for the first time as the perception to which a person is perceived as a “real person” to relate to others in the process of using the media to communicate. Social presence is not only an attribute of the media but also a psychological perception formed by participants (Gunawardena and Zittle, 1997). In electronic supplier websites, social presence affects purchase behavior (Gefen et al., 2003). The use of rich descriptions and pictures in commercial websites can increase social presence (Hassanein and Head, 2007; Chin et al., 2021b). Generally, social presence refers to the feeling of being with others, and the interactivity generated by social presence can influence users’ behavior to purchase virtual products (Jin et al., 2017). Existing research on social presence mainly focuses on the above aspects, ignoring its role in the field of medical crowdfunding. Therefore, it is necessary to examine how social presence affects crowdfunding behavior.

The potential donors among social media users mainly learn about the detailed content of the patients by browsing the text words of donation instructions, pictures and videos released by donation platforms, etc. Detailed introduction to project content and video presentations can make donors feel high quality (Bi et al., 2017). Therefore, patients need to provide more detailed information on the website, such as health status, economic conditions, area and other content characteristics, in order to meet the content quality requirements of the donor group (Duan et al., 2021). Since the social presence can penetrate websites through rich text descriptions, pictures, and other different project descriptions, the higher the richness and completeness of the content, the more it can affect the social presence (Hassanein and Head, 2007). Based on the above analysis, this paper believes that the detailed disclosure of self-information by patients in medical crowdfunding, such as the severity of disease, whether economic conditions are difficult, and whether the area is poor or not, affects the social presence. Therefore, it proposes the following hypotheses in the paper:

*Hypothesis 1*: The richness of project descriptions has a positive effect on social presence.

*Hypothesis 2*: The completeness of the patient status display has a positive effect on social presence.

The diffusion of medical crowdfunding projects is mainly through internet applications, and most of the donors are social media users. Users can interact through media, and this interaction and participation can be directly measured by likes, comments, and forwards (Cho et al., 2014; Hong et al., 2018). User participation can not only promote cognition and emotion among related donors, and improve the state of participation, but also enable donors to generate corresponding relationships or interactive behaviors through this state (Robiady et al., 2021). This paper argues that the interactive behaviors generated by users participating in medical crowdfunding can stimulate social presence. Therefore, it proposes the following hypotheses in the paper:

*Hypothesis 3*: User participation has a positive effect on the social presence.

Individual donation behavior is often referred to as the public welfare consumption behavior. Therefore, crowdfunding donors, as a special kind of consumers, also have a sense of social presence in the process of participating in crowdfunding. This paper believes that the social presence of medical crowdfunding consists of two aspects, the perception of the psychological reality of the patient when users browse the project information released by the donation platform through media such as network platforms, and the perception of the psychological reality of other donors through online interaction. That is to say, it is the psychological perception that users can produce a kind of presence with others in media such as network platforms. This perception of social presence can promote the generation of donation behavior by perceiving the presence of others (Bereczkei et al., 2010). Therefore, it proposes the following hypotheses in the paper:

*Hypothesis 4*: Social presence has a positive effect on donation behavior.

### Perceived differences in trust

The psychologist Deutsch (1958) first proposed the study of trust in the prisoner’s dilemma experiment. As a description of people’s psychological state perception, trust has differentiated characteristics. Perceived differences in trust show that the degree of trust is heterogeneous (Huang et al., 2022).

In the context of medical crowdfunding, the drive of trust influences investment behaviors (Kang et al., 2016). In terms of factors affecting the credibility and success of crowdfunding projects, performance, emotion, and perceived differences in trust all influence crowdfunding investors’ investment behavior (Liang et al., 2019). Perceived differences in trust and project quality are important factors affecting trust in crowdfunding platforms (Moysidou and Hausberg, 2020). Cognitive and emotional trust has significant effects on donation behavior (Aleksina et al., 2019; Zhang et al., 2021b). Therefore, it proposes the following hypotheses in the paper:

*Hypothesis* 5: Perceived differences in trust have a negative effect on donation behavior.

In general, potential donors to medical crowdfunding need to carefully check the quality of the project and judge its credibility before deciding to donate. When the quality information displayed by the platform institutions is limited, for example, the donors do not know how the funds are used, etc., it will cause the supporters of donation to have a sense of distrust (Prakash and Gugerty, 2010). Furthermore, project quality is related to the success of a crowdfunding project (Mollick, 2014). Projects with lower quality and transparency performed worse in perceived trust and donation behavior (Bekkers and Wiepking, 2011). Based on the above analysis, this paper believes that in the context of medical crowdfunding, project quality can be analyzed through the project description and project transparency. From the perspective of project transparency, the platform conducts fundraising dynamic updates and fund announcements in time, sending high-quality and trustworthy signals to the public, to reduce perceived differences in trust (Saxton et al., 2012). Therefore, it proposes the following hypotheses in the paper:

*Hypothesis 6*: Project transparency has a negative effect on perceived differences in trust.

It is worth mentioning that since there is an effect between empathy and trust (Colquitt et al., 2007), empathy can increase trust (Lupoli et al., 2020). Women, children, and disabled persons, as vulnerable groups in society, should be cared for and sympathized with by other members of society. This paper argues that once such vulnerable groups appear on medical crowdfunding platforms as patients, they will arouse people’s sympathy and trust. Therefore, it proposes the following hypotheses in the paper:

*Hypothesis 7*: The vulnerability of the patient’s identity has a negative effect on perceived differences in trust.

### S-O-R theoretical model

The S-O-R (Stimulus-Organism-Response) theory, which originated from environmental psychology, was proposed by environmental psychologists Mehrabian and Russell (1974) based on the further development of the S-R model, to explain and predict different environmental stimuli to affect human cognition and emotion to affect individual behavior. Researchers introduced it into the study of online consumer behavior for the first time and believed that the consumer’s sense of atmosphere would affect consumer behavior (Eroglu et al., 2001). In exploring how website quality affects consumers’ purchase behavior through the two variables, Chang and Chen (2008) used S-O-R theory to take perceived trust and risk as mediating variables. The researchers used it to study the purchase behavior of social commerce websites (Liu et al., 2019; Zhang et al., 2020).

Based on the above studies, it can be found that many scholars have confirmed that many online behaviors of individuals follow the dynamic process of stimulus-organism-response based on the SOR theory. Therefore, based on this theoretical model, this paper combs the psychological mechanism of Stimulus (Project description, Project transparency, Patient status, User participation, Patient identity)-Organism (social presence, perceived differences in trust)-Response (donation behavior), and verifies the mediating roles of the social presence and perceived differences in trust, and then analyzes the individuals’ donation behavior in the field of medical crowdfunding.

**Figure 1:**
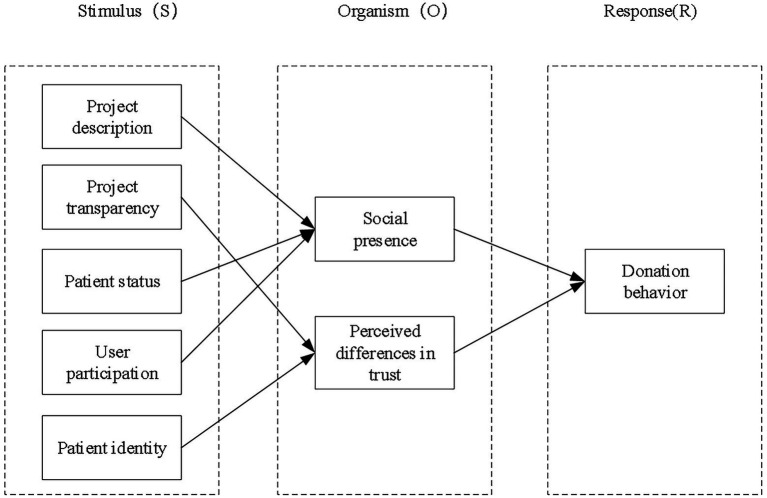
Research model of donation behavior for medical crowdfunding projects.

According to the above hypotheses and the S-O-R theory, the research model of this paper is proposed, as shown in Figure 1.

## Research method

### Research approach

The empirical research approach is adopted in the research to test the proposed research hypothesis. The data used in the empirical research are collected from the questionnaire. The reason why we use this research approach is that it is a popular method, and its conclusions on the phenomena studied are objective and can be tested according to experience and facts.

### Samples and data

This paper will collect data by means of a questionnaire survey. The distributed questionnaire mainly includes two parts: demographic variables and variable questions. When the questionnaires were officially distributed, a total of 497 questionnaires were distributed. After excluding invalid questionnaires, 437 valid questionnaires were obtained, with a questionnaire return rate of 87.93%.

### Measurement of variables

The measurement question items of the relevant variables in this paper are set based on the existing research at home and abroad, referring to the relevant literature in the field of medical crowdfunding, and making appropriate modifications in combination with the research questions. The variables were measured using the Likert 5 score scale, with 1 for a strong disagreement and 5 for strong agreement. The measuring items of variables in this study are shown in Table 1.

**Table 1:** Measuring items of variables.

Latent variable name and code	Measurement question item
Project description (DE)	DE1 I am very concerned about whether the text description can infect me.
	DE2 The more pictures and videos, the more it can inspire my sympathy.
	DE3 I am more attracted to well-described medical crowdfunding projects.
Project transparency (TR)	TR1 The timely update of the fundraising dynamic enables me to understand the progress of the project.
	TR2 The timely announcement of funds can make me believe in the authenticity of this project.
	TR3 I think the more transparent the project, the more I can trust.
Patient status (ST)	ST1 The more serious the disease of the patient, the more urgent the situation, the more I have the idea of helping.
	ST2 The fact that the patient discloses his or her financial distress allows me to know what kind of help the patient needs.
	ST3 The poorer the area where the patient lives, the more I have the idea of helping.
User participation (US)	US1 Interacting with others can lead me to the idea of joining the donation team.
	US2 It makes me feel more authentic when I see personal comments from other donors, or descriptions related to their condition provided in the comments.
	US3 I will interact on WeChat or Moments by liking, commenting, or forwarding.
Patient identity (ID)	ID1 I’m more willing to trust women than men.
	ID2 I think younger patients need help more than older ones.
	ID3 I think the disabled or incapacitated patients really need help.
Social presence (PR)	PR1 Reading the comments someone has just posted while browsing the donation page makes me perceive the presence of other donors.
	PR2 I can feel the real situation of the patient through the detailed information.
	PR3 When I donate online, I feel like my actions are largely detectable by others.
Perceived differences in trust (PE)	PE1 I do not think that the platform I follow is necessarily trustworthy.
	PE2 I do not think that the information released by the patient I am concerned about is necessarily worthy of my trust.
	PE3 I do not think the information released by the platform I follow is necessarily true.
Donation intention (DO)	DO1 I really want to help those who are in trouble.
	DO2 I plan to help those who are in trouble.
	DO3 I will suggest to my relatives and friends to help the patient I am concerned about.

## Research results and analysis

### Reliability and validity analysis

This paper uses SPSS 25.0 and AMOS 25.0 to analyze the questionnaire data to verify the consistency and validity of the measurement scale. According to the test results of the scale, the overall KMO test value is 0.937, the factor loading of each item of all variables is above 0.7, the Cronbach’s coefficient of each variable is above 0.7, the CR value is all above 0.7, and most of the AVE values are above 0.5, indicating that the scale has a good internal consistency and good convergent validity. The specific test results are shown in Table 2.

**Table 2:** Reliability and validity test results.

Variables	Question item	Standard factor loading	Cronbach’s a	CR	AVE
Project description (DE)	DE1	0.657	0.790	0.792	0.565
	DE2	0.790			
	DE3	0.793			
Project transparency (TR)	TR1	0.708	0.811	0.817	0.600
	TR2	0.853			
	TR3	0.756			
Patient status (ST)	ST1	0.689	0.752	0.788	0.554
	ST2	0.742			
	ST3	0.698			
User participation (US)	US1	0.639	0.764	0.772	0.532
	US2	0.795			
	US3	0.745			
Patient identity (ID)	ID1	0.651	0.740	0.749	0.500
	ID2	0.781			
	ID3	0.683			
Social presence (PR)	PR1	0.696	0.743	0.735	0.482
	PR2	0.743			
	PR3	0.639			
Perceived differences in trust (PE)	PE1	0.807	0.859	0.860	0.673
	PE2	0.843			
	PE3	0.810			
Donation behavior (DO)	DO1	0.664	0.830	0.795	0.565
	DO2	0.801			
	DO3	0.783			

In this paper, we also use multiple linear regression to further test the multicollinearity. The results show that the Durbin-Watson test value is 2.049 and there is no autocorrelation, and the VIF value of each variable is less than the critical value of 10, which indicates that there is no serious multicollinearity problem in this study.

### Model evaluation and hypothesis test

In the analysis based on reliability and validity, the paper uses AMOS 25.0 to build a structural equation model for confirmatory factor analysis. The results are shown in Table 3, which lists the results of the structural equation model fitting index analysis in this paper. The fitting values of the model are all within the acceptable range. Therefore, the model in this paper is of good fitness.

**Table 3:** Fit index of this model.

	CMIN/DF	RMSEA	GFI	NFI	IFI	AGFI	CFI
Acceptable range	<5	<0.08	>0.8	>0.8	>0.8	>0.8	>0.8
Model fitting index	4.478	0.072	0.844	0.811	0.846	0.805	0.845

In this paper, under the premise of acceptable model fitness, the hypothesis of the model is tested, and the specific results are shown in Table 4. Among them, project description and user participation have a significant positive effect on social presence, and hypotheses H1 and H3 are established. Project transparency, patient identity and social presence have a negative effect on perceived differences in trust. Social presence has a positive effect on donation behavior. Perceived differences in trust also have a negative effect on donation behavior. Hence, hypotheses H4, H5, H6 and H7 are supported.

**Table 4:** Test results of the standardized path analysis.

Hypothesis	Path	Estimate	SE	*t*-value	*p*	Test result
H1	DE → PR	0.194^*^	0.082	2.555	0.011	Support
H2	ST → PR	0.040	0.118	0.337	0.736	Not support
H3	TR → PR	0.698^***^	0.133	4.962	^***^	Support
H4	PR → DO	0.472^***^	0.079	5.682	^***^	Support
H5	PE → DO	−0.569^***^	0.065	−7.387	^***^	Support
H6	US→PE	−0.509^***^	0.06	−9.045	^***^	Support
H7	ID→PE	−0.421^***^	0.063	−7.477	^***^	Support

* *p* < 0.05 and

*** *p* < 0.001.

In addition, from the analysis results, it can be seen that the patient status does not have a significant effect on the social presence, and the hypothesis H2 is not supported.

### Analysis of mediating effect

This paper uses the Bootstrap function of AMOS 25.0 to conduct 5,000 repeated samplings with the confidence interval of 95% to test whether the perceived differences in trust and social presence generated by users in the process of participating in medical crowdfunding can play a mediating role. The analysis results are shown in Table 5. Under the confidence interval of 95%, according to the judgment standard that the upper and lower intervals of bias-corrected and percentile do not contain 0, and the value Z ≥ 1.96 is taken as the establishment of the indirect effect. The indirect effect of the four paths is significant (Z ≥ 1.96), the direct effect is also significant (Z ≥ 1.96), and the trust interval does not contain 0, indicating that social presence can partially mediate the behavior to donate through project description and user participation, and perceived differences in trust can partially mediate the behavior to donate through project transparency and patient identity.

**Table 5:** Test Results of the bootstrap mediating effect.

Path	Effect	Point Estimation	Product of Coefficients		Bootstrap 5,000 Times					
					Bias-corrected			Percentile		
			SE	Z	Lower	Upper	*p*-Value	Lower	Upper	*p*-Value
DE → PR → DO	Indirect effect	0.416	0.108	3.852	0.248	0.685	0.000	0.242	0.666	0.000
	Direct effect	0.345	0.127	2.717	0.096	0.599	0.011	0.095	0.597	0.012
	Total effect	0.761	0.098	7.765	0.575	0.963	0.000	0.577	0.968	0.000
TR → PR → DO	Indirect effect	0.369	0.169	2.183	0.093	0.775	0.021	0.037	0.706	0.038
	Direct effect	0.506	0.221	2.290	0.144	1.007	0.013	0.137	0.994	0.015
	Total effect	0.875	0.116	7.543	0.673	1.133	0.000	0.666	1.120	0.000
US→ PE → DO	Indirect effect	0.426	0.09	4.733	0.274	0.639	0.000	0.259	0.615	0.000
	Direct effect	0.308	0.15	2.053	0.07	0.611	0.011	0.068	0.658	0.012
	Total effect	0.734	0.166	4.422	0.485	1.149	0.000	0.475	1.126	0.000
ID→ PE → DO	Indirect effect	0.509	0.096	5.302	0.354	0.737	0.000	0.341	0.715	0.000
	Direct effect	0.256	0.11	2.327	0.051	0.489	0.017	0.045	0.483	0.019
	Total effect	0.764	0.096	7.958	0.595	0.972	0.000	0.588	0.964	0.000

## Discussion

### Research conclusion

This paper explores the internal mechanism of social presence and perceived differences in trust under medical crowdfunding. The following conclusions were drawn from this study.

Firstly, social presence plays a mediating role between project description and donation behavior, and between user participation and donation behavior. The project description has a significant positive effect on the social presence, which shows that the medical crowdfunding website can make the visitor feel high quality and promote the generation of social presence by displaying rich text descriptions and pictures. Patient status has no significant effect on social presence. The reason may be that once the patient releases too much information about their own content, it will lead to an overload of browsing user information, which will not only affect their understanding of the information content, but also make them feel that their browsing freedom is restricted. The higher the user participation, the more it can promote the generation of social presence. This shows that in the Internet environment, the interaction and participation behaviors of social media users will enhance the perception of connection and existence between each other, which is conducive to the formation of social presence.

Secondly, perceived differences in trust play a mediating role between project transparency and donation behavior, and between patient identity and donation behavior. The more transparent the project, the more it reduces perceived differences in trust and promotes perceived trust. This shows that the timely update of the fundraising dynamic and timely announcement of funds on the medical crowdfunding platform enable donors to have a comprehensive understanding of the project and enhance their trust. The patient identity has a negative effect on perceived differences in trust, indicating that compared with the general group, vulnerable groups can arouse the sympathy and trust of potential donors.

Finally, in terms of influencing the donation behavior, social presence has a significant positive effect on the donation behavior, but perceived differences in trust have a significant negative effect. The results of this study are consistent with the conclusions of Zhang et al. (2021b) in different contexts. Empirical results show that the social presence created in medical crowdfunding and perceived differences in trust formed by users in the process of medical crowdfunding can have an indirect effect on donation behavior. This shows that the establishment of a strong trust relationship and a full sense of social presence are conducive to the transformation of donation behavior by various factors in the medical crowdfunding scenario.

### Research implications

#### Theoretical contributions

Based on the S-O-R research framework, this paper extends social presence and perceived differences in trust to the medical crowdfunding context. Moreover, compared with previous direct relationship studies, this paper constructs an intermediary effect model through the social presence and perceived differences in trust to deeply study the influence mechanism of donation behavior of medical crowdfunding projects. While exploring the influencing factors and mechanisms of donation behavior, it not only confirms the significant effect of social presence and perceived differences in trust on donation behavior, but also finds the mediating roles of them, which expands the application of these theories.

#### Practical implications

On the one hand, the medical crowdfunding platform should focus on reducing perceived differences in trust and enhancing perceived trust. These platforms can pay attention to the disclosure of the project fundraising process and the use of funds while supervising and reviewing the project, and must fully display the high-quality project content to ensure that the visitors have a multi-faceted understanding and cognition of the project. On the other hand, Platform operators should focus on creating a social presence on the platform. And should pay attention to users’ needs for interaction, guide and establish a good communication and interaction mechanism between users, and continuously develop and optimize the interactive mode of the comment area to promote the improvement of users’ social presence. This can not only provide potential donors with more clues of cognition, but also make them feel more emotional value and a sense of substitution while alleviating their concerns, and stimulate a stronger sense of social presence, in order to promote the donors’ donation behavior and enhance the vitality of the relevant crowdfunding industry.

## Data availability statement

The original contributions presented in the study are included in the article/Supplementary material; further inquiries can be directed to the corresponding author.

## Author contributions

TZ and QZ designed the research and wrote the manuscript. RJ and TG collected the data and offered modification suggestions. MY provided guidance throughout the entire research process. All authors contributed to the article and approved the submitted version.

## Funding

This research was supported by National Natural Science Foundation of China (nos. 11861071 and 71462036), Yunnan Fundamental Research Projects (nos. 202101AT070211 and 202201AT070142), and School-level Project of Yunnan University of Finance and Economics (no. 2021B02).

## Conflict of interest

The authors declare that the research was conducted in the absence of any commercial or financial relationships that could be construed as a potential conflict of interest.

## Publisher’s note

All claims expressed in this article are solely those of the authors and do not necessarily represent those of their affiliated organizations, or those of the publisher, the editors and the reviewers. Any product that may be evaluated in this article, or claim that may be made by its manufacturer, is not guaranteed or endorsed by the publisher.
